# Supplementary material for: Determinants of SARS-CoV-2 Infection in the Older Adult Population: Data from the LOST in Lombardia Study
Source: Vaccines (Basel). 2022 Jun 22;10(7):989. doi: 10.3390/vaccines10070989 (PMC9324825; doi:10.3390/vaccines10070989)
Supplement: Supplementary file 1 [file vaccines-10-00989-s001.zip › vaccines-1763748-supplementary.pdf]

**Supplementary Table S1**: Distribution of 4400 Italian subjects aged 65 years or more according to COVID-19 infection and type of diagnosis, overall and by sex and age. LOST IN LOMBARDIA, 2020.

| Characteristics | Total | Sex | | Age | | | | |
|---|---|---|---|---|---|---|---|---|
| | | Men | Women | 65-69 | 70-74 | 75-79 | 80-84 | 85+ |
| Total | 4400 (100) | 1902 (100) | 2498 (100) | 1289 (100) | 838 (100) | 1188 (100) | 739 (100) | 346 (100) |
| COVID-19 | | | | | | | | |
| No | 4187 (95.2) | 1807 (95.0) | 2379 (95.3) | 1195 (92.7) | 811 (96.8) | 1136 (95.7) | 707 (95.6) | 338 (97.7) |
| Yes | 213 (4.9) | 95 (5.0) | 119 (4.8) | 94 (7.3) | 27 (3.2) | 51 (4.3) | 33 (4.4) | 8 (2.3) |
| Type of diagnosis* | | | | | | | | |
| Swab | 98 (45.8) | 49 (51.4) | 49 (41.3) | 46 (49.1) | 15 (53.9) | 18 (35.9) | 15 (46.8) | 3 (38.3) |
| Serological test | 46 (21.6) | 18 (19.5) | 28 (23.3) | 24 (25.5) | 4 (15.7) | 14 (26.9) | 4 (12.0) | 0 (0.0) |
| No diagnosis, symptoms only | 70 (32.7) | 28 (29.1) | 42 (35.5) | 24 (25.4) | 8 (30.5) | 19 (37.2) | 14 (41.2) | 5 (61.7) |

*on subjects with COVID-19
